# Severe pulmonary hypertension in a young patient with end-stage renal disease on chronic hemodialysis

**DOI:** 10.4103/0974-2069.74055

**Published:** 2010

**Authors:** Satyavan Sharma, Ashok L Kirpalani, Amit Kulkarni

**Affiliations:** Department of Cardiology, Bombay Hospital and Medical Research Centre, Mumbai, Maharashtra, India

**Keywords:** Arteriovenous fistula, end-stage renal disease, pulmonary artery hypertension, pulmonary circulation

## Abstract

Severe pulmonary hypertension in a teenager with end-stage renal disease on chronic hemodialysis via arteriovenous access is reported. Clinical presentation included persistent volume overload and pericardial effusion. Serial hemodynamic data obtained at cardiac catheterization confirmed the diagnosis. In addition, detailed biochemical and imaging data (echo- Doppler, computed tomography of chest, computed tomographic pulmonary angiography, VQ lung scan, etc.) were obtained to find out the mechanism. The exact cause of pulmonary hypertension remains unclear, and a multi- factorial mechanism is postulated. This rare case is presented to highlight the role of aggressive dialysis, pericardiocentesis, and use of sildenafil and bosentan in the management.

## INTRODUCTION

Pulmonary hypertension (PHT) is defined as an elevation of pulmonary artery pressure secondary to heart, lung or systemic disorders. PHT is a well-known, though uncommon, complication of end-stage renal disease (ESRD).[1] The pathogenesis of PHT in this group of patients remains unclear. It can result from vasoconstriction and obliteration of lumen of small vessels in the lung by plexiform lesions, resulting in increased resistance to flow.[2] Hormonal and metabolic derangements associated with ESRD might lead to pulmonary arterial vasoconstriction and increase in pulmonary vascular resistance.[3] PHT can result in extremely serious morbidity and reduced survival.[4] Early detection of the disease is necessary to prevent development of significant patho-physiological changes.

We report a young patient with ESRD on chronic hemodialysis, who developed severe PHT. The diagnosis and management modalities of this potentially lethal condition are discussed.

## CASE REPORT

A 15 year old male was referred for cardiac evaluation for worsening dyspnea and orthopnea for the last 2 weeks. The patient was on maintenance hemodialysis for the last 6 years owing to ESRD. Clinical examination revealed tachycardia, patent arteriovenous access on left arm, markedly elevated- jugular- venous pressure with prominent a and v waves, blood pressure of 130/80 mm Hg and clear lung fields. The patient had radio- cephalic fistula constructed at the left wrist between the radial artery and the cephalic vein, using end to end anastomosis. Cardiac assessment revealed a large heart, and loud pulmonic component of the second heart sound without any murmurs or rub. Electrocardiogram was unremarkable. Skiagram of the chest revealed a cardiothoracic ratio of 0.75, prominent right heart border, prominent superior caval vein and clear lung fields.

Relevant blood investigations showed elevated serum creatinine 5.8 mg/dl (normal 0.3–0.9 mg/dl), low hemoglobin 9.5 g/dl (normal 12–16 g/dl), low serum calcium 8 mg/dl (normal 9.5–11 mg/dl), marginally elevated serum parathormone 67 pg/ml (normal 15–65 pg/ml), elevated phosphorus 6.8 mg/dl (normal 2.5–5.9 mg/dl), calcium–phosphorus product 54.4 mg^2^/dl^2^, total protein 6.3 g/dl (normal 6.4–8.2 g/dl), albumin 3.4 g/dl (normal 3.4–5.0 g/dl), globulin 2.9 g/dl (normal 2.5–4.0 g/dl) and albumin/globulin (A/G ratio) 1.01 (normal 0.9–2).

Cross-sectional echocardiography with Doppler interrogation revealed large pericardial effusion without any tamponade, normal cardiac valves without calcification or regurgitation. There was no evidence of any intra or extra cardiac shunt. Right atrium and right ventricle were dilated with evidence of severe PHT. There was moderate tricuspid regurgitation with well-preserved right ventricular function. Systolic pulmonary artery pressure was estimated to be 110 mm Hg on continuous wave Doppler. Left ventricular ejection fraction was 60% and there was no evidence of left ventricular diastolic dysfunction. Further investigations were performed to evaluate the cause of PHT. Complete pulmonary function tests were normal. Radioisotope lung scan showed no evidence of ventilation-perfusion mismatch. Computed tomography of the chest confirmed a large pericardial effusion, and right heart dilatation, and revealed bilateral ground glass densities with interlobular thickening, suggesting pulmonary edema. Computed tomographic pulmonary angiography showed dilated main pulmonary trunk, right and left pulmonary arteries without any evidence of thromboembolism.

Hemodynamic data obtained at right and left heart catheterization is summarized in Table 1. The oxygen saturation was normal while breathing room air. Left ventriculography revealed normal left ventricular ejection fraction, no valvular regurgitation and ruled out cardiomyopathy. Coronary vessels were normal on angiography. Pericardiocentesis yielded 150 ml of hemorrhagic fluid and an essentially unchanged hemodynamics. Pericardial fluid did not show any evidence of tuberculosis, autoimmune disorder, infection or malignancy. The patient was advised aggressive dialysis protocol (4 hours on alternate days) and oral sildenafil (60 mg daily in three divided doses). Other treatment included alpha-methyl dopa 250 mg twice a day, calcium and folic acid supplementation.

Follow up at 8 months revealed marked symptomatic benefit and improved effort tolerance. He could walk for 6 minutes on treadmill on modified Bruce protocol. Hemodynamic data were obtained at right and left heart catheterization [Table 2] to assess the progress and serve as a baseline data for endothelin-receptor antagonist, bosentan therapy. Pulmonary artery pressure recorded at right heart catheterization was nearly similar to the one obtained by Doppler interrogation. Bosentan therapy (current dosage of 62.5 mg twice a day) for the last 3 months produced further symptomatic benefit and reduction in pulmonary artery pressure to 70 mm Hg on Doppler. Follow-up right and left heart catheterization is scheduled after 9 months, i.e., after 1 year of bosentan therapy. The end point for bosentan and sildenafil therapy will be either any side effect precluding their use, normalization of pulmonary artery pressure or renal transplant. The patient is awaiting renal transplantation.

**Table 1:** Hemodynamic data at cardiac catheterization

Site/variables	Pressure (mm Hg)	
	Basal	After pericardiocentesis
SCV	a- 28, v- 20; mean 24	a- 20, v- 16; mean 16
RA	a- 28, v- 20; mean 24	a- 20, v- 16; mean 16
RV	104/20	90/16
MPT	104/50; mean 68	90/40; mean 60
Pulmonary wedge	Mean 20	Mean 14
Aorta	120/80; mean 100	120/80; mean 100
LV	120/20	120/14
Cardiac output in l/min (thermodilution)	6.5	6.8
PVR (woods unit)	7.3	5
SVR (woods unit)	14.7	9.7
Pericardial pressure	12	3

SCV, superior caval vein; RA, right atrium; RV, right ventricle; MPT, main pulmonary trunk; LV, left ventricle; PVR, pulmonary vascular resistance; SVR, systemic vascular resistance

**Table 2:** Hemodynamic data at cardiac catheterization prior to Bosentan therapy

Site/variables	Pressure (mm Hg)	
RA	a- 24, v- 20, mean 22
RV	100/50 with mean 70
PW	Mean 14
FA	110/70; mean 80
Cardiac output in l/min	6.8
PVR (wood unit)	8.2
SVR (wood unit)	7

FA, femoral artery, RA, right atrium; RV, right ventricle; MPT, main pulmonary trunk; PW, Pulmonary wedge

## DISCUSSION

During the fourth world symposium on PHT held in 2008 at Dana point, California, experts proposed an updated classification of PHT.[5] This classification mentions PHT with unclear multi-factorial mechanisms to occur in patients with ESRD on long-term hemodialysis.[5] Based on echocardiographic study,[6] pulmonary hypertension is well documented in patients with ESRD receiving long-term hemodialysis with surgical arteriovenous access. The reported patients are mostly in the fourth or fifth decades of life, wherein ESRDhas resulted from hypertension or diabetes.[7] The occurrence of severe degree of PHT in a young patient is rare and prompted us to report the case. There are several potential explanations for the development of PHT in these patients. Hormonal and metabolic derangement associated with end-stage renal disease might lead to pulmonary vascular constriction. Mazdeh and colleagues[4] found significantly low hemoglobin and low albumin in middle-aged patients with PHT in their study. These parameters were marginally low in the present case and it is difficult to comment on their significance from a single case. The pulmonary artery pressure can also increase due to high cardiac output (resulting from the arteriovenous access itself and often concomitant anemia) as well as fluid overload.[8] Studies on the role of parathyroid hormone have provided conflicting data.[1,9] The duration of arteriovenous fistula and flow through it have been correlated with high incidence of PHT.[9] Diastolic and systolic dysfunction of the left ventricle frequently seen in renal failure can also contribute to pulmonary hypertension.[10]

In this young patient, detailed investigations ruled out any apparent cause for the severe PHT. It seems that PHT is multi- factorial and the major contribution is from the increased cardiac output due to arteriovenous fistula. Pathological elevation of pulmonary artery pressure occurs in those patients in whom pulmonary circulation cannot compensate for arteriovenous access related elevated cardiac output. There is a high prevalence of pulmonary hypertension among patients with ESRD in pre-dialysis period after creation of arteriovenous fistula and on chronic hemodialysis via a surgical arteriovenous fistula.[11] It has been recently postulated that patients with ESRD have endothelial dysfunction of pulmonary circulation.[10] Anemia, fluid overload and metabolic factors seem to have played a minor role. The development of PHT is associated with increased mortality and morbidity. Management is challenging and involves early renal transplantation, reducing the size of arteriovenous fistula, or change over to other modes of dialysis without arteriovenous access. Sildenafil, a phosphodiesterase-5-inhibitor has been used extensively in patients with PHT due to a variety of conditions. It improved exercise tolerance and symptomatic status in this young patient. Faraz and colleagues[12] reported a case of PHT on hemodialysis, who had significant clinical and hemodynamic improvement following the use of endothelin- receptor antagonist, bosentan. This case report prompted us to use this agent with encouraging preliminary results. Several questions regarding the dose, duration of sildenafil and bosentan and their likely effect on hemodialysis and renal transplantation will emerge. Larger studies and longer follow- up of this patient will help in answering some of these issues.
